# A randomised phase III trial comparing gemcitabine with surgery-only in patients with resected pancreatic cancer: Japanese Study Group of Adjuvant Therapy for Pancreatic Cancer

**DOI:** 10.1038/sj.bjc.6605256

**Published:** 2009-08-18

**Authors:** H Ueno, T Kosuge, Y Matsuyama, J Yamamoto, A Nakao, S Egawa, R Doi, M Monden, T Hatori, M Tanaka, M Shimada, K Kanemitsu

**Affiliations:** 1 Hepatobiliary and Pancreatic Oncology Division, National Cancer Center Hospital, Tokyo, Japan; 2 Hepatobiliary and Pancreatic Surgery Division, National Cancer Center Hospital, Tokyo, Japan; 3 Department of Biostatistics, Tokyo University, Tokyo, Japan; 4 Department of Gastrointestinal Surgery, Cancer Institute Hospital, Tokyo, Japan; 5 Department of Surgery II, Nagoya University, Aichi, Japan; 6 Division of Gastroenterology, Tohoku University, Miyagi, Japan; 7 Department of Surgery, Kyoto University, Kyoto, Japan; 8 Department of Surgery and Clinical Oncology, Osaka University, Osaka, Japan; 9 Department of Surgery, Tokyo Women's Medical University, Tokyo, Japan; 10 Department of Surgery and Oncology, Kyushu University, Fukuoka, Japan; 11 Department of Surgery, Tokushima University, Tokushima, Japan; 12 Department of Gastroenterological Surgery, Kumamoto University, Kumamoto, Japan

**Keywords:** pancreatic cancer, phase III, adjuvant chemotherapy, gemcitabine

## Abstract

**Background:**

This multicentre randomised phase III trial was designed to determine whether adjuvant chemotherapy with gemcitabine improves the outcomes of patients with resected pancreatic cancer.

**Methods:**

Eligibility criteria included macroscopically curative resection of invasive ductal carcinoma of the pancreas and no earlier radiation or chemotherapy. Patients were randomly assigned at a 1 : 1 ratio to either the gemcitabine group or the surgery-only group. Patients assigned to the gemcitabine group received gemcitabine at a dose of 1000 mg m^−2^ over 30 min on days 1, 8 and 15, every 4 weeks for 3 cycles.

**Results:**

Between April 2002 and March 2005, 119 patients were enrolled in this study. Among them, 118 were eligible and analysable (58 in the gemcitabine group and 60 in the surgery-only group). Both groups were well balanced in terms of baseline characteristics. Although heamatological toxicity was frequently observed in the gemcitabine group, most toxicities were transient, and grade 3 or 4 non-heamatological toxicity was rare. Patients in the gemcitabine group showed significantly longer disease-free survival (DFS) than those in the surgery-only group (median DFS, 11.4*versus* 5.0 months; hazard ratio=0.60 (95% confidence interval (CI): 0.40–0.89); *P*=0.01), although overall survival did not differ significantly between the gemcitabine and surgery-only groups (median overall survival, 22.3 *versus* 18.4 months; hazard ratio=0.77 (95% CI: 0.51–1.14); *P*=0.19).

**Conclusion:**

The current results suggest that adjuvant gemcitabine contributes to prolonged DFS in patients undergoing macroscopically curative resection of pancreatic cancer.

Pancreatic cancer remains to be one of the most challenging malignancies to treat. Surgical resection offers the only opportunity for cure. However, as no valid method for early detection of this disease has been established, 80% or more of patients present with unresectable disease at the time of diagnosis. Furthermore, even when resection is performed, the recurrence rate is extremely high, resulting in the 5-year survival rate of patients with resected pancreatic cancer being no more than 20% (Evans *et al*, 1997). As surgical resection alone has limitations, development of non-surgical treatments, including adjuvant therapy, is needed to improve the prognosis of patients with pancreatic cancer.

Several previous studies have suggested the efficacy of adjuvant chemoradiotherapy and/or chemotherapy for the treatment of resected pancreatic cancer (Kalser and Ellenberg, 1985; Neoptolemos *et al*, 2004; Stocken *et al*, 2005; Hazard *et al*, 2007). In the United States, adjuvant chemoradiation with fluorouracil has become the standard of care after the Gastrointestinal Tumour Study Group study showed a statistically significant improvement in survival as compared with surgery-only (median overall survival, 20 *versus* 11 months; 2-year survival rate, 42 *versus* 15%) (Kalser and Ellenberg, 1985). Recently, an evaluation of Medicare patients derived from the SEER database showed a survival advantage for patients who received adjuvant chemoradiotherapy as compared with patients who did not (3-year survival rate, 45 *versus* 30%) (Hazard *et al*, 2007). On the other hand, no survival benefit of adjuvant chemoradiation was shown by the European Study Group for Pancreatic Cancer (ESPAC)-1 trial, a large-scale phase III study conducted in Europe (Neoptolemos *et al*, 2004). In the ESPAC-1, chemotherapy using fluorouracil plus leucovorin, but not chemoradiation, showed efficacy in the adjuvant setting: for patients who received postoperative chemotherapy compared with those who did not, the 2-year survival rates (40 *versus* 30%) and the 5-year survival rates (21 *versus* 8%) were significantly greater. Therefore, although the benefit of adjuvant therapy has become more apparent in recent years, the optimal treatment modality remains controversial (Ueno and Kosuge, 2008; Zuckerman and Ryan, 2008).

As for unresectable advanced pancreatic cancer, gemcitabine has been widely employed, since Burris *et al* (1997) reported results of a phase III study. The results of this study suggested that patients receiving gemcitabine experienced improved survival as compared with those receiving fluorouracil (median overall survival, 5.65*versus* 4.42 months; *P*=0.0025). The efficacy and tolerability of gemcitabine for advanced pancreatic cancer have been confirmed by several subsequent studies (Berlin *et al*, 2002; Moore *et al*, 2003; Rocha Lima *et al*, 2004), and gemcitabine has become the standard therapy for unresectable pancreatic cancer. These facts led investigators to evaluate gemcitabine in the adjuvant setting for patients with resected pancreatic cancer.

In 2005, a large phase III study, CONKO-001 (Charité Onkologie), was presented at the American Society of Clinical Oncology (ASCO) Annual Meeting by a German group (Oettle *et al*, 2007). CONKO-001 compared a gemcitabine therapy group with a surgery-only group after macroscopically curative resection of pancreatic cancer. In CONKO-001, disease-free survival (DFS) was significantly longer in the gemcitabine than in the observation group (median DFS, 13.4 *versus* 6.9 months; *P*<0.001). However, overall survival did not differ significantly between the gemcitabine and surgery-only groups, although the survival period tended to be longer in the gemcitabine than in the observation group (median, 22.1 *versus* 20.2 months; *P*=0.06).

Coincidentally, at approximately the same time as the CONKO-001, our multicentre randomised phase III trial, JSAP-02 (Japanese Study Group of Adjuvant Therapy for Pancreatic Cancer), was being conducted to test whether the addition of adjuvant gemcitabine to surgery would improve the outcomes of patients with resected pancreatic cancer. The JSAP-02 study design basically resembled that of CONKO-001, except for the planned number of gemcitabine cycles: six cycles of gemcitabine were used in CONKO-001 and three cycles in our study. To our knowledge, this is the first randomised phase III trial of adjuvant gemcitabine in an Asian population.

## Patients and methods

### Trial design

JSAP-02 was conducted at 10 centres in Japan. The trial was supported by funding from the Health and Labour Sciences Research Grant for Clinical Cancer Research from the Ministry of Health, Labour and Welfare, Japan.

The primary end point was overall survival. Secondary end points were DFS and gemcitabine safety. The ethics boards of all institutions approved the protocol and all patients provided a written, informed consent. The trial was conducted in accordance with the World Medical Association Declaration of Helsinki and Japanese Good Clinical Practice guidelines. The trial was monitored for excessive toxicity by the Data Monitoring Committee, which functions independently of the JSAP. Data were collected using the web-based clinical trial management system at the data centre (EPS Co., Ltd., Osaka, Japan), and additional changes were locked out of the database on 31 March 2009.

### Patient eligibility

Patients who underwent macroscopically curative resection of pancreatic cancer were enrolled in the study 3 to 10 weeks after surgery. The other eligibility criteria were histologically proven invasive ductal carcinoma of the pancreas; no history of earlier chemotherapy or radiotherapy for pancreatic cancer except intra-operative radiotherapy; age 20–74 years; Karnofsky performance status of 50 or more; and adequate organ function (WBC count ⩾4000 and ⩽12 000 mm^−3^; neutrophil count ⩾2000 mm^−3^; platelet count ⩾100 000 mm^−3^; haemoglobin level ⩾9.0 g per 100 ml; serum total bilirubin level ⩽3.0 mg per 100 ml; serum aspartate aminotransferase and serum alanine aminotransferase level ⩽5 times the upper limit of the normal range; and serum creatinine level lesser than or equal to the upper limit of the normal range). The exclusion criteria were pulmonary fibrosis or interstitial pneumonia; clinically significant pleural effusions; presence of distant metastasis (except distant lymph node metastasis confirmed by resected specimen); other concomitant malignant disease; active infection; history of serious complications related to surgery; active gastrointestinal ulcers; history of myocardial infarction within 3 months; severe mental disorder; pregnant or lactating women; and other serious concomitant systemic disorders incompatible with the trial in the investigator's judgment.

### Treatment plan

Patients were enrolled, within 10 weeks after surgery, through fax by the staff at the data centre. Patients were randomly assigned at a 1 : 1 ratio to either the gemcitabine group or the surgery-only group using the minimisation method stratified by resection status (R0 *versus* R1), pathological stage (I–II *versus* III–IV) and enrollment centre. Stage classification and the evaluation of resected specimens were performed in accordance with the fifth edition of the tumour–node–metastasis classification system of the International Union Against Cancer. Patients assigned to the gemcitabine group received gemcitabine at a dose of 1000 mg m^−2^ over 30 min on days 1, 8 and 15 every 4 weeks. This 4-week cycle was repeated for 3 cycles. If patients developed leukocyte counts of <2000 mm^−3^ or >12 000 mm^−3^, or platelet counts of <75 000 mm^−3^ during chemotherapy, gemcitabine administration was stopped until recovery. When patients had grade 4 leukopenia or neutropenia, febrile neutropenia or infection with grade 3 leukopenia or neutropenia, a platelet count of <25 000 mm^−3^, or non-heamatological toxic effects of grade 3 or greater, a dose reduction of gemcitabine from 1000 mg m^−2^ to 800 mg m^−2^ was allowed. The surgery-only group received no anticancer treatment after surgery, unless there was a confirmed relapse.

### Assessments

Baseline assessments included medical history, physical examination, vital signs, chest radiography, ECG, routine laboratory tests, and the tumour markers CEA and CA19-9. Patients in the gemcitabine group underwent laboratory tests and assessment of clinical symptoms every week during the treatment period and every 3 months after completing adjuvant chemotherapy. Patients in the surgery-only group underwent similar examinations every 3 months. Adverse events were assessed according to the Common Toxicity Criteria of the National Cancer Institute (version 2.0). Patients in both groups underwent computed tomography and/or ultrasonography at 3-month intervals after surgery, unless there was a confirmed relapse. Tumour markers, CEA and CA19-9, were also measured every 3 months until relapse.

### Statistical analysis

A total of 116 patients were required to detect a hazard ratio of 0.55 with 80% power at a two-sided 0.05 significance level, which corresponds to a 20% increase in the 2-year overall survival rate in the surgery-only group *versus* the gemcitabine group (15 *versus* 35%, respectively).

All randomised and eligible patients were included in the intent-to-treat (ITT) population for efficacy analyses. Efficacy analyses were also performed in subpopulations stratified by resection status (R0 *versus* R1) and pathological stage (I–II *versus* III–IV). For safety analyses of gemcitabine, only patients who received adjuvant gemcitabine were included. Overall survival was defined as the period between randomisation and death. All deaths, including those from other diseases, were considered to be events. Disease-free survival was defined as the period between randomisation and the occurrence of an event—relapse or death—whichever came first. Data for patients who had not had an event were censored, as of the date of the final observation. The Kaplan–Meier method was used to estimate the overall survival or DFS and the log-rank test was used for comparisons between the two groups. The Wilcoxon test, Fisher's exact test and the Mantel trend test were used to compare differences among pretreatment characteristics between the two groups. *P*-values of less than 0.05 were considered to indicate statistical significance. All statistical analyses were performed using SAS version 9.1 statistical software (SAS Institute Inc, Cary, NC, USA).

## Results

### Characteristics of patients

**Figure 1:**
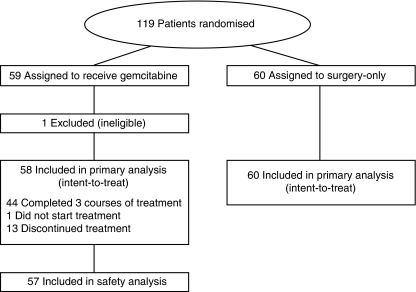
Flow chart of study subjects.

Between April 2002 and March 2005, 119 patients in total were enrolled at 10 centres. After randomisation, one patient in the gemcitabine group was found to be ineligible because of a low WBC count at baseline. Therefore, 118 eligible patients (58 in the gemcitabine group and 60 in the surgery-only group) were included in the ITT population for efficacy analyses (Figure 1). No patients assigned to the surgery-only group received postoperative anticancer treatment until a confirmed relapse. The two groups were well balanced with regard to baseline characteristics (Table 1). In total, 16% of the patients had a microscopically positive margin (R1) and 69% had nodal metastases (N1). The median follow-up period for surviving patients was 60.4 months (range, 40.6–77.1 months) on the analysis cut-off date of 31 March 2009.

### Treatment administration

Among the 58 patients in the gemcitabine group, one withdrew from the study before treatment because of a postoperative complication. Six patients (10%) discontinued treatment within 1 cycle, 7 (12%) after 2 cycles and 44 (76%) completed the scheduled 3 cycles of treatment. The reasons for withdrawal from treatment included adverse events or complications (10 patients), the detection of recurrent disease (2 patients) and patient preference (2 patients). The dose of gemcitabine was decreased in one patient because of neutropenia. The median number of cycles and the median number of gemcitabine doses administered were 3 and 8, respectively. The median dose intensity of gemcitabine was 667 mg m^−2^ per week, and the median relative dose intensity was 89%.

### Safety

Of the 58 eligible patients assigned to the gemcitabine group, adverse events were analysed in 57 patients who received at least one dose of gemcitabine. Major grade 3 or 4 adverse events observed during the treatment are listed in Table 2. Adjuvant gemcitabine was generally well tolerated. Although high frequencies of grade 3 or 4 leukopenia and neutropenia were experienced (25 and 70%, respectively), most myelosuppression resolved promptly without complications.

Three fatal events occurred during the study period: two in the gemcitabine group and one in the surgery-only group. Of the two, an association with gemcitabine could not be ruled out in one patient who developed an abdominal abscess without neutropenia after two treatment cycles and died from gastrointestinal bleed 183 days after the final gemcitabine administration.

### DFS and overall survival

**Figure 2:**
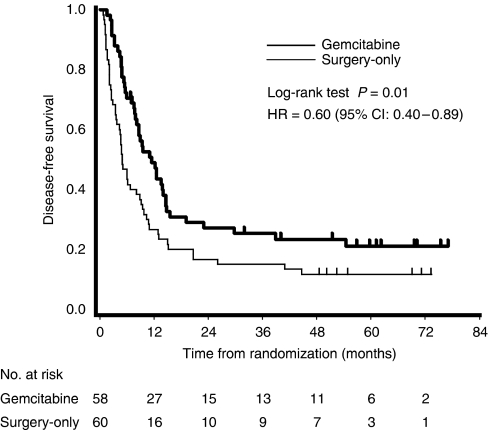
Kaplan–Meier estimates of disease-free survival. Intent-to-treat analysis.

**Figure 3:**
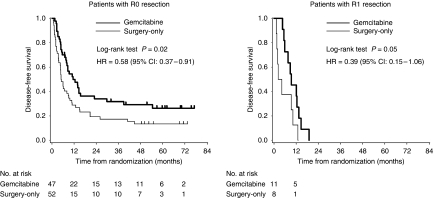
Kaplan–Meier estimates of disease-free survival in patients with R0 or R1 resection. Intent-to-treat analysis.

At the time of analysis, 44 patients in the gemcitabine group and 53 in the surgery-only group had recurrent disease. The common sites of first recurrence were the liver, peritoneum and local recurrence (Table 3). The recurrence pattern was similar in the two groups. DFS was significantly longer in the gemcitabine group than in the surgery-only group, with an estimated hazard ratio of 0.60 (95% confidence interval (CI), 0.40–0.89; *P*=0.01; Figure 2). Median DFS was 11.4 months (95% CI, 8.0–14.5) in the gemcitabine group *versus* 5.0 months (95% CI, 3.7–8.9) in the surgery-only group. The estimated DFS rates at 6, 12 and 24 months were 70.7, 49.0 and 27.2% in the gemcitabine group, and 43.3, 26.7 and 16.7% in the surgery-only group, respectively. Subgroup analyses showed that the beneficial effect of adjuvant gemcitabine on DFS was evident for R0, N0 and stage I–II patients (Table 4, Figure 3).

**Figure 4:**
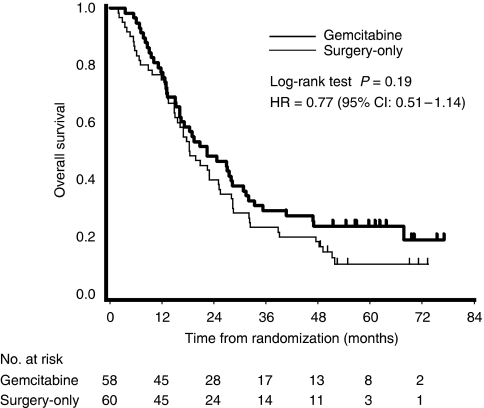
Kaplan–Meier estimates of overall survival. Intent-to-treat analysis.

At the time of analysis, 98 patients (83%) had died (45 patients in the gemcitabine group and 53 in the surgery-only group). The causes of death in the gemcitabine group and surgery-only groups were as follows: relapse (41 and 52 patients, respectively), adverse events (2 and 1 patients, respectively) and unknown causes (2 and 0 patients, respectively). Log-rank analysis revealed no statistically significant difference in survival estimates between the treatment groups (hazard ratio, 0.77 (95% CI, 0.51–1.14); *P*=0.19; Figure 4). Median overall survival was 22.3 months in the gemcitabine group (95% CI, 16.1–30.7) *versus* 18.4 months in the surgery-only group (95% CI, 15.1–25.3). The estimated overall survival rates at 6, 12, 18, 24 and 60 months were 94.8, 77.6, 58.6, 48.3 and 23.9% in the gemcitabine group, and 85.0, 75.0, 53.3, 40.0 and 10.6% in the surgery-only group, respectively. Subgroup analyses failed to show the beneficial effect of adjuvant gemcitabine on overall survival, although the survival period tended to be longer in the gemcitabine than in the observation group for R0, N0 and stage I–II patients (Table 4).

## Discussion

We found that DFS in patients with resected pancreatic cancer was significantly improved with three cycles of adjuvant gemcitabine as compared with surgery-only, with an estimated hazard ratio of 0.60 (*P*=0.01). However, a statistically significant improvement in overall survival was not shown in this study, although median overall survival, and 2-year and 5-year survival rates were favourable in the gemcitabine group as compared with the surgery-only group. These results were similar to those of the previously reported phase III trial of adjuvant gemcitabine, CONKO-001 (Oettle *et al*, 2007).

CONKO-001 compared six cycles of gemcitabine with surgery-only after macroscopically curative resection of pancreatic cancer. Table 5 shows a comparison of our study (JSAP-02) and CONKO-001. The study design of JSAP-02 basically resembled that of CONKO-001 except for the planned sample size, number of gemcitabine cycles, weeks from surgery to randomisation and eligibility criteria determined by postoperative tumour markers. Baseline patient characteristics, including resection status and nodal status, were similar between the two studies. As for efficacies, although both studies failed to show a statistically significant improvement in overall survival, a significantly better DFS was shown with the adjuvant gemcitabine. The data on DFS and overall survival reported in JSAP-02 were comparable with those in CONKO-001.

The CONKO-001 data were re-analysed in March 2008, and presented at the ASCO Annual Meeting of that year as the final results (Neuhaus *et al*, 2008). Although the improvement in overall survival did not reach statistical significance (*P*=0.06) in the previous report (Oettle *et al*, 2007), the new CONKO-001 report showed a significant difference in overall survival between the gemcitabine and surgery-only groups after long-term observation (median overall survival, 22.3 months *versus* 20.2 months; 5-year survival rate, 21.0% *versus* 9.0%; *P*=0.005). In contrast to these final results of CONKO-001, our study, though the data were analysed after an adequate observation period, failed to show a survival benefit of adjuvant gemcitabine. As the JSAP-02 survival curve itself resembled that of CONKO-001, the main reason for this discrepancy may be the underpowered nature of our study. The planned sample size for the JSAP-02, which was less than one-third that of CONKO-001, might have been too small to detect a significant difference in overall survival. Other factors that differed between the two studies, including race, number of gemcitabine cycles and patient selection based on postoperative tumour markers, may also have influenced the outcome of our study. Further study is needed to clarify the impacts of these factors on adjuvant gemcitabine. Although intra-operative radiotherapy was allowed only in our study and 52% of patients actually received this treatment, its influence may be very small because a recent phase III trial failed to show any benefits of intra-operative radiotherapy in patients with resected pancreatic cancer (Kinoshita *et al*, 2009).

Adjuvant gemcitabine was well tolerated in our study. A total of 44 patients (76%) completed the three scheduled treatment cycles, and the median relative dose intensity of gemcitabine was as good as 89%. Although 70% of patients experienced grade 3 or 4 neutropenia during adjuvant gemcitabine therapy, most of these toxicities were transient, and serious adverse events were rare. The frequencies of grade 3 or 4 neutropenia induced by gemcitabine monotherapy are reportedly 20–30% (Aapro *et al*, 1998). The reasons for marked heamatological toxicities occurring in our study are unclear, although surgical stress might have exacerbated myelosuppression. Onoue *et al* (2004) reported that administering gemcitabine to patients after surgical resection resulted in more severe leukopenia, as compared with patients not undergoing resection (grade 3 or 4 leukopenia, 57 *versus* 25%; *P*=0.048). Although our study, similar to CONKO-001, showed the safety of adjuvant gemcitabine, cautious selection of patients and careful observation of treatment will be necessary when giving this agent to patients with resected pancreatic cancer.

Other than gemcitabine, fluorouracil-based chemotherapy is now considered to be an option for adjuvant therapy for resected pancreatic cancer based on the results of ESPAC-1. The ESPAC-1 study showed a survival benefit of adjuvant fluorouracil plus leucovorin in 289 patients with resected pancreatic cancer (Neoptolemos *et al*, 2004). The ESPAC group also performed a pooled analysis using data from 458 patients who were enrolled in ESPAC-1, ESPAC-1 plus or early ESPAC-3(v1) (Neoptolemos *et al*, 2009a). The overall survival was superior in patients randomised to fluorouracil plus leucovorin, as compared with those randomised to observation (pooled hazard ratio, 0.70; *P*=0.003, median overall survival, 23.2 *versus* 16.8 months), indicating the validity of using fluorouracil plus leucovorin as adjuvant therapy.

With regard to the comparison between gemcitabine and fluorouracil-based chemotherapy in the adjuvant setting, two large phase III trials, RTOG 97-04 and ESPAC-3(v2), were recently reported (Regine *et al*, 2008; Neoptolemos *et al*, 2009b). RTOG 97-04 examined whether survival could be extended by substituting gemcitabine for fluorouracil before and after fluorouracil-based radiation. When the data from the entire population were analysed, no significant difference in the survival period was noted between the fluorouracil and gemcitabine groups, but the gemcitabine group had better outcomes when the analysis was confined to patients with pancreatic head cancer (median overall survival, 20.5 *versus* 16.9 months, *P*=0.033). ESPAC-3(v2) was designed to compare fluorouracil plus leucovorin and gemcitabine in patients with resected pancreatic cancer. In total, 1088 patients were randomised in ESPAC-3(v2), and Neoptolemos *et al* reported no significant difference in survival between adjuvant fluorouracil plus leucovorin and adjuvant gemcitabine at the 2009 ASCO Annual Meeting (hazard ratio, 0.94; *P*=0.39, median overall survival 23.0 *versus* 23.6 months). Although no significant difference in survival was shown, gemcitabine may be suitable for clinical use as adjuvant therapy because the rate of serious adverse events in patients treated with gemcitabine was significantly lower than that in patients treated with fluorouracil plus leucovorin (7.5 *versus* 14%, *P*<0.001).

In recent years, new approaches, including novel cytotoxic or molecular-targeting agents, have been actively applied in the adjuvant setting for pancreatic cancer. In Japan, S-1, an oral fluoropyrimidine derivative, has attracted the attention of investigators on the basis the promising results of clinical trials for advanced pancreatic cancer (Ueno *et al*, 2007; Okusaka *et al*, 2008). We are now conducting a phase I/II trial of gemcitabine plus S-1 for resected pancreatic cancer (JSAP-03 trial). As well as developing new effective treatments, individualised approaches based on individual differences in drug metabolism are also important in selecting patients who are more likely to benefit from adjuvant gemcitabine. Several recent studies have suggested that tumour-specific expression of human equilibrative nucleoside transporter 1 may be a promising predictive biomarker of outcome in pancreatic cancer patients receiving gemcitabine chemotherapy (Spratlin *et al*, 2004; Giovannetti *et al*, 2006; Farrell *et al*, 2009; Maréchal *et al*, 2009). Further investigation of and progress in these new strategies are expected in the future.

In conclusion, adjuvant chemotherapy with gemcitabine significantly improved DFS, as compared with surgery-only in patients with resected pancreatic cancer. Our study supports the conclusions of the CONKO-001 as well as the validity of using gemcitabine as adjuvant therapy for resected pancreatic cancer.

## Figures and Tables

**Table 1:** Baseline characteristics

	**Gemcitabine (*n*=58)**			**Surgery-only (*n*=60)**			***P*-value**
**Characteristic**	**No.**		**%**	**No.**		**%**	
*Age (years)*							
Median		65			64		0.62
Range		41–74			36–74		
							
*Sex*							
Women	18		31	26		43	0.19
Men	40		69	34		57	
							
*Days from surgery to randomisation (days)*							
Median		44			47		0.45
Range		22–71			22–70		
							
*Karnofsky performance status*							
Median		90			90		0.83
Range		70–100			70–100		
							
*Intra-operative radiotherapy*							
Yes	27		47	34		57	0.36
No	31		53	26		43	
							
*Primary site*							
Head	42		72	42		70	0.84
Body-tail	16		28	18		30	
							
*Maximal tumour size (cm)*							
Median		3.5			3.5		0.27
Range		1.0–10.0			1.2–7.0		
							
*Resection status*							
R0	47		81	52		87	0.46
R1	11		19	8		13	
							
*Primary tumour size*							
T1	6		10	6		10	0.90
T2	1		2	4		6	
T3	31		53	28		47	
T4	20		35	22		37	
							
*Nodal status*							
N0	19		33	18		30	0.84
N1	39		67	42		70	
							
*Pathological stage* a							
I	3		5	4		7	0.82
II	10		17	10		17	
III	21		36	22		37	
IV	24		41	24		40	
							
*Grading*							
1	18		31	16		27	0.80
2	33		57	36		60	
3	5		9	4		7	
Unknown	2		3	4		7	
							
*Histology*							
Adenocarcinoma	56		97	56		93	0.68
Other	2		3	4		7	
							
*CEA (ng ml* ^*−1*^ *)*							
Median		3.7			4.6		0.34
Range		0.9–252			0.5–74		
							
*CA19–9 (U ml* ^*−1*^ *)*							
Median		33.2			37.5		0.40
Range		0–10 435			0–46 100		

Abbreviations: CEA=carcinoembryonic antigen; CA 19–9=carbohydrate antigen 19–9.

a UICC fifth edition.

**Table 2:** Grade 3 and 4 adverse events in the gemcitabine group (*n*=57)

	**Gemcitabine**			
	**Grade 3** a		**Grade 4** a	
**Adverse event**	**No.**	**%**	**No.**	**%**
*Heamatological*				
Leukopenia	13	23	1	2
Neutropenia	32	56	8	14
Anaemia	2	4	0	0
Thrombocytopenia	1	2	0	0
				
*Non-heamatological*				
Diarrhoea	1	2	0	0
Fever	1	2	0	0
Nausea	0	0	1	2
Anorexia	1	2	1	2
Fatigue	1	2	0	0
AST	3	5	0	0
ALT	4	7	0	0
Abscess	0	0	1	2

Abbreviations: ALT=alanine aminotransferase; AST=aspartate aminotransferase.

a NCI Common Toxicity Criteria, version 2.0.

**Table 3:** Patterns of initial recurrence

	**Gemcitabine**		**Surgery-only**	
	**No.**	**%**	**No.**	**%**
Local	10	23	17	32
Liver	13	30	16	30
Peritoneum	8	18	7	13
Other	12	27	12	23
Unknown	1	2	1	2

**Table 4:** Disease-free and overall survivals in the total entire population and subgroups

			**Disease-free survival**				**Overall survival**			
	**No.**		**Median (months)**				**Median (months)**			
	**GEM**	**Surgery-only**	**GEM**	**Surgery-only**	**HR (95% CI)**	***P*-value**	**GEM**	**Surgery-only**	**HR (95% CI)**	***P*-value**
All patients	58	60	11.4	5.0	0.60 (0.40–0.89)	0.01	22.3	18.4	0.77 (0.51–1.14)	0.19
R0	47	52	11.4	5.1	0.58 (0.37–0.91)	0.02	26.8	19.1	0.70 (0.45–1.09)	0.11
R1	11	8	9.5	3.4	0.39 (0.15–1.06)	0.05	18.3	17.6	1.05 (0.41–2.72)	0.92
N0	19	18	—	9.0	0.38 (0.16–0.86)	0.02	32.0	28.4	0.63 (0.29–1.37)	0.24
N1	39	42	8.6	4.5	0.73 (0.46–1.16)	0.19	17.1	17.3	0.84 (0.53–1.34)	0.84
Stage I–II	13	14	—	10.0	0.27 (0.08–0.85)	0.02	67.8	—	0.42 (0.15–1.22)	0.10
Stage III–IV	45	46	8.9	4.6	0.68 (0.44–1.05)	0.08	18.3	16.3	0.82 (0.53–1.26)	0.36

Abbreviations: CI=confidence interval; GEM=gemcitabine; HR=hazard ratio.

**Table 5:** Comparison between the current Japanese study and CONKO-001

	**Current study (JSAP-02)**			**CONKO-001** a		
	**Gemcitabine**		**Surgery-only**	**Gemcitabine**		**Surgery-only**
*Study design*						
Planned sample size		116			368	
Planned number of gemcitabine cycles	3		—	6		—
Planned weeks from surgery to randomisation		⩽10			⩽6	
Selection based on postoperative tumour markers		No requirement			⩽2.5 times the upper limit of normal	
						
*Baseline patient characteristics*						
No. of patients	58		60	179		175
Median age (years)	65		64	62		61
Sex: men	69%		57%	59%		56%
Median Karnofsky PS	90		90	80		80
Resection status: R0	81%		87%	81%		85%
Nodal status: N0	33%		30%	29%		27%
Median days from surgery to randomisation	44		47	22		24
						
*Results*						
Median DFS (months)	11.4		5.0	13.4		6.9
1-year DFS rate	49%		27%	58%		31%
2-year DFS rate	27%		17%	31%		15%
*P*-value		0.01			<0.01	
Median OS (months)	22.3		18.4	22.1		20.2
1-year OS rate	78%		75%	73%		73%
2-year OS rate	48%		40%	48%		42%
5-year OS rate	24%		11%	23%		12%
*P*-value		0.19			0.06	

Abbreviations: DRF=disease-free survival; OS=overall survival; PS=performance status.

a Previously reported in Oettle *et al* (2007).
